# The Interplay Between Rheumatoid Arthritis and Pregnancy Outcomes: A Narrative Review

**DOI:** 10.7759/cureus.105178

**Published:** 2026-03-13

**Authors:** Nilam Mishra, Karishma Bal, Bishnupriya Lenka, Monalisa Mall, Anasuya Behera, Barsharani Behera, Rojalin Dash, Debashree Kar, Pritilagna Dash

**Affiliations:** 1 Obstetrics and Gynaecology, Kalinga Institute of Nursing Sciences (KINS), Bhubaneswar, IND; 2 Nursing, Kalinga Institute of Nursing Sciences (KINS), Bhubaneswar, IND; 3 Obstetrics and Gynaecology Nursing, Kalinga Institute of Nursing Sciences (KINS), Bhubaneswar, IND; 4 Obstetrics and Gynaecology, Kalinga Institute of Nursing Sciences, Kalinga Institute of Industrial Technology Deemed to be University (KIIT-DU), Bhubaneswar, IND; 5 Obstetrics and Nursing, Kalinga Institute of Nursing Sciences (KINS), Bhubaneswar, IND; 6 Community Health Nursing, Kalinga Institute of Nursing Sciences (KINS), Bhubaneswar, IND; 7 Medical Surgical Nursing, Kalinga institute of Nursing Sciences, Kalinga Institute of Industrial Technology Deemed to be University (KIIT-DU), Bhubaneswar, IND

**Keywords:** autoimmune disease, disease-modifying therapies, fetal health, immunological changes, maternal health, preconception counselling, pregnancy complications, pregnancy outcomes, ra medications, rheumatoid arthritis

## Abstract

Rheumatoid arthritis (RA) is a chronic autoimmune disease that commonly affects women during their reproductive years. With increasing use of disease-modifying therapies and greater emphasis on pregnancy planning, it is essential to understand how RA and its treatment impact pregnancy outcomes.

This narrative review explores the pathophysiological changes of RA during pregnancy, evaluates maternal and fetal outcomes, examines the safety of RA medications, and highlights the importance of preconception counseling and coordinated care. A range of studies published between 2015 and 2025, including cohort studies, population-based registries, and prospective clinical research, were reviewed. These studies addressed immunological and hormonal changes during pregnancy, obstetric and neonatal risks, and safety profiles of various RA treatments.

Pregnancy induces immune and hormonal shifts that often lead to reduced RA symptoms in the second and third trimesters, though disease flares are common after delivery. Despite improved therapies, women with RA remain at higher risk for complications such as miscarriage, preeclampsia, gestational diabetes, and cesarean delivery. Infants born to mothers with RA are at increased risk of preterm birth, low birth weight, and small-for-gestational-age status. Certain medications, like methotrexate, are unsafe in pregnancy and should be stopped in advance, while others, such as hydroxychloroquine and tumor necrosis factor (TNF) inhibitors, are considered relatively safe. Although RA presents significant challenges during pregnancy, favorable outcomes are possible through preconception planning, disease control, and multidisciplinary care. Continued research and personalized treatment strategies are essential to improve reproductive outcomes in women with RA.

## Introduction and background

Connective tissue diseases (CTD) are systemic disorders affecting the body’s support tissues, divided into hereditary and autoimmune types. Autoimmune CTDs encompass a range of disorders that can affect various organs in the body, including systemic lupus erythematosus (SLE), systemic sclerosis (SSc), and rheumatoid arthritis (RA), which affect women during their reproductive years [1]. RA is a chronic inflammatory disorder with a prevalence of approximately 1% in the general population, and it can affect women of reproductive age. In recent decades, there has been significant development of new medications, including biologic disease-modifying antirheumatic drugs (bDMARDs) and Janus kinase (JAK) inhibitors. With these advancements, the use of glucocorticoids has decreased, and more patients are achieving remission in the current era [2].

Preconception care is an essential aspect of future family planning, particularly for patients with RA, for several reasons. First, teratogenic medications are sometimes used in RA management. Second, patients with RA are more prone to infertility, and lastly, RA disease activity itself can impact pregnancy outcomes [3]. Neonates born to mothers with RA had a twofold increase in the risk for Low Birth Weight and prematurity (LBW)and were 1.7 times more likely to be small for gestational age [4]. Pregnant women with RA experienced antepartum hemorrhage (APH) and required CS more often than those without RA [4].

Subfertility is a common problem in women with RA. Approximately 40% of female patients with RA suffer from subfertility (a time to pregnancy (TTP) longer than 12 months despite regular unprotected intercourse) compared with 10%-15% of women from the general population [5]. The frequency of APO in RA patients was 41.5%, 18.3% experienced spontaneous abortions, 11.0% underwent preterm deliveries, 7.3% had small for gestational age infants, 4.9% experienced intrauterine growth restriction, 1.2% experienced stillbirth, and 1.2% suffered from eclampsia [6]. Older females (>35 years old) had a higher probability of having a pregnancy with complications, consistent with data from general pregnant populations 

Research results showed that miscarriages, preterm deliveries, small for gestational age (SGA) infants, and intrauterine growth restriction were the most common complications [6].

A recent meta-analysis of 237 pregnancies in RA patients, using objective scores of disease activity, has demonstrated that only 60% of patients improve in disease activity during pregnancy, and 50% experience worsening symptoms in the postpartum period [7].

This narrative review aims to synthesize current evidence on the pathophysiology of RA during pregnancy, its impact on maternal and fetal outcomes, and the importance of preconception counseling and disease management to optimize reproductive health in women with RA.

The management of RA patients wishing to become pregnant involves adaptation of the therapeutic regimen, thereby balancing the need to withdraw teratogenic drugs, such as MTX and LEF, while keeping disease activity under control. In the rheumatological setting, data are available on the use of anti-TNF therapy in the preconception period or during pregnancy in women with RA [8].

Women of reproductive age and can significantly influence fertility and family planning decisions. These conditions not only impose disease-related burdens but may also be compounded by concerns about pharmacologic therapy. Biologic disease - modifying antirheumatic drugs (bDMARDs) represent an important therapeutic advancement, offering targeted immunomodulation through agents such as tumor necrosis factor (TNF) inhibitors, anti-CD20 antibodies, interleukin blockers, and T-cell modulators [9,10].

Women with rheumatoid arthritis (RA) often experience reproductive challenges, with subfertility reported in approximately 46% of cases, frequently due to anovulation or unexplained causes. Periconceptional use of NSAIDs is associated with impaired ovulation. Delayed conception is common, as active disease necessitates treatment with teratogenic drugs, leading to prolonged disease activity and potential joint damage. Many women with RA report reduced family size or cessation of conception efforts due to disease activity or contraindicated medications. These findings highlight the importance of preconception counseling, optimized disease control, and multidisciplinary care to improve reproductive outcomes in RA patients [11].

## Review

The search methodology for this narrative review followed a comprehensive and structured approach to identify relevant literature. The review utilized two commonly used databases, PubMed and Web of Science. Search terms for the review included ‘Rheumatoid arthritis’, ‘pregnancy outcomes’, ‘fetal outcomes’, ‘impact of medications of RA on pregnancy outcomes’, along with related keywords, combined using Boolean operators to refine and optimize search results.

The study aims to give clear guidance on planning a pregnancy for women who have RA. It focuses on how to reduce health risks for both the mother and the baby and how to achieve the best possible pregnancy outcomes. The study explains how doctors should check how active the disease is, review medicines to make sure they are safe for pregnancy, and look for other health problems that might affect getting pregnant or carrying a baby. It also highlights the importance of teamwork between the rheumatologist, obstetrician, and other specialists to make a personalized care plan. By encouraging proper family planning, safe contraception, and keeping RA under good control before getting pregnant, the study aims to help women with RA have safer pregnancies and healthy babies, similar to women without RA.

A total of 29 studies based on relevance, quality, and methodological precision were included for the narrative review. These studies analyzed the Interplay Between RA and Pregnancy Outcomes.

The exact etiology of increased subfertility rates in women with RA remains unclear, but it is likely multifactorial. For example, some women with RA may delay pregnancy due to disease activity or teratogenic medication use. There can be psychosocial stressors that contribute to subfertility in RA as well, such as concerns about disease flaring during or after pregnancy, concerns about functional limitations in caring for a child, and/or stress about the heritability of RA to their offspring. Few women (8%) reported being advised to limit family size, although approximately 20% reported that RA had affected their childbearing decisions [12].

RA can also be characterized by disease-specific autoantibodies, including rheumatoid factor (RF) and anti-cyclic citrullinated peptide (anti-CCP). These antibodies are used to distinguish “seropositive” and “seronegative” forms of RA, and there is ongoing debate regarding the direct impact of these antibodies on the pathogenesis of RA. Prednisone and sulfasalazine were most frequently prescribed during pregnancy [13]. Tumor necrosis factor inhibitors (TNFi) have become an important component of the treatment of rheumatic diseases during pregnancy. A drawback of prescribing TNFi during pregnancy involves active transport of these drugs across the placenta mediated by neonatal Fc receptors (FcRn) [14]. Placental transfer starts around gestational week 20, and the rate of transfer increases throughout pregnancy [15].

The treatment of RA has evolved: early diagnosis, immediate initiation of disease- modifying antirheumatic drugs (DMARDs), several new approved drugs, and a treat- to- target (T2T) approach aiming for remission have resulted in better outcomes for patients [14].

TNFi have revolutionized the treatment of RA. Data suggest that use of TNF inhibitors is not associated with effects on conception or teratogenic risk [15]. Treatment with TNF inhibitors and/or a combination of DMARDs is considered a key element of a T2T approach [13,16].

Research analysis showed that in pregnant women with RA, there was a significantly increased risk of adverse maternal outcomes, including cesarean section (95%), preeclampsia (95%), gestational hypertension (95%), and spontaneous abortion (95% ) [16]. Studies have indicated that female patients with RA have a high rate of planned pregnancies. Similarly, maternal RA during pregnancy was also associated with a significantly increased risk of adverse fetal outcomes, including preterm birth (95%), small for gestational age (95%), low birth weight (95%), congenital anomalies (95%), and stillbirth (95%). Arthritis worsened after delivery for half of the women with RA. During pregnancy, just over half of women with RA were taking medications, the most common being TNF inhibitors or prednisone [17]. Unexplained subfertility (48%) and anovulation (28%) were the most common gynecologic diagnoses, and both occurred more often in RA patients than reported in the general population. Women with unexplained subfertility more often used nonsteroidal anti-inflammatory drugs (NSAIDs) during the peri-conceptional period [18]. 42-46% of women with RA suffer from low fertility [19,20]. Women with RA have more difficulty conceiving than women without RA [19]. Women with active RA are more likely to have preterm births, small-for-gestational-age babies, and require medical interventions during delivery [21]. In terms of maternal outcomes, there was an increased rate of cesarean delivery, preeclampsia, and preterm delivery in RA patients [22].

The management of RA during pregnancy requires careful consideration due to the potential teratogenicity and safety profiles of commonly used disease-modifying antirheumatic drugs (DMARDs) and biologics. Methotrexate (MTX), a cornerstone of RA therapy, is strongly contraindicated during pregnancy, with studies reporting a significantly increased risk of pregnancy loss and elective terminations when exposure occurs within the three months prior to conception. The risk reduction for RA in parous women correlated with maternal age and was shown to be significant for women younger than 45 years of age [23]. Hydroxychloroquine (HCQ), in contrast, is generally considered safe and may be continued throughout pregnancy due to its favorable fetal safety profile [24]. The use of corticosteroids and biologics, particularly TNFi, has also been explored; TNF inhibitors appear to be relatively safe and may be continued during at least the first half of pregnancy to maintain disease remission [25]. High disease activity itself, especially when requiring combination therapy or steroids, has been associated with adverse outcomes, including preeclampsia and low birth weight [26]. Consequently, preconception counseling and appropriate timing of drug discontinuation, particularly for teratogenic agents like MTX, are essential to minimize risks and support favorable pregnancy outcomes [27].

Fetal and neonatal outcomes in pregnancies complicated by RA reveal a heightened risk of adverse events compared to the general population. Multiple studies have consistently reported increased rates of preterm birth, intrauterine growth restriction (IUGR), and low birth weight (LBW) among women with RA [28]. For instance, Gupta et al. noted significantly lower average birth weights and higher incidences of complicated deliveries in RA patients compared to healthy controls. Similarly, a study found an elevated risk of preeclampsia and associated neonatal complications, especially in cases with high maternal disease activity or combination therapy exposure, further contributing to early deliveries and NICU admissions [29]. Though congenital anomalies are not universally elevated in all studies, the potential for neonatal complications, including NICU stays and even stillbirths, was reported in cohorts with uncontrolled disease or exposure to certain medications, such as methotrexate. These findings underscore the critical importance of preconception planning, tight disease control, and multidisciplinary monitoring throughout pregnancy to optimize fetal and neonatal health outcomes.

RA during pregnancy can increase risks such as preterm birth, low birth weight, and delivery complications, potentially affecting the child’s immediate and long-term health. Active RA may also impair the mother’s ability to care for her newborn due to fatigue, pain, or postpartum flares, impacting bonding and maternal well-being. Poor pregnancy outcomes may have lasting effects on the child’s development. Early intervention, disease control, and coordinated care are essential to improve outcomes for both mother and baby (Table 1).

**Table 1:** Features of reviewed articles

Author	Year	Key Findings	Conclusion
Smeele et al. [13]	2021	Modern treatment led to low disease activity in 90% of pregnant rheumatoid arthritis patients (PreCARA-Pre conception Counseling in Active Rheumatoid Arthritis).	Aggressive treatment yields positive pregnancy outcomes in rheumatoid arthritis.
Simister and Story [14]	1997	Explained the role of Fragment Crystallizable receptors in antibody transfer during pregnancy.	Understanding immune transfer helps in evaluating fetal safety of biologics.
Smolen et al. [15]	2016	Updated treatment-to-target guidelines for rheumatoid arthritis management.	Tight disease control improves outcomes, including in pregnant women.
Huang et al. [16]	2023	Maternal RA is significantly associated with an increased risk of adverse maternal and fetal outcomes.	Close monitoring during pregnancy is crucial for women with Rheumatoid Arthritis.
Brouwer et al. [18]	2017	Assessed subfertility and fertility treatment outcomes in women with rheumatoid arthritis.	Rheumatoid arthritis can lead to subfertility; referral to fertility services may be necessary.
De Jong and Dolhain [19]	2017	Reviewed fertility, pregnancy, and lactation in rheumatoid arthritis.	Personalized care throughout reproductive stages enhances outcomes.
Al Rayes et al. [20]	2021	Found higher rates of miscarriage and complications in rheumatoid arthritis pregnancies in Saudi Arabia.	Rheumatoid arthritis adversely affects pregnancy; region-specific data support global findings.
Sim et. al [21]	2023	Systematic review and meta-analysis confirmed poorer pregnancy outcomes in Rheumatoid Arthritis.	Rheumatoid arthritis pregnancy management requires multidisciplinary approach.
Zanetti et al. [22]	2022	Found methotrexate associated with increased miscarriage risk.	Avoiding teratogenic drugs like methotrexate before conception is essential.
Barbhaiya et al. [23]	2013	Reviewed management of rheumatoid arthritis and systemic lupus erythematosus during pregnancy.	Evidence-based management is needed for optimal maternal-fetal outcomes.
Østensen et al. [24]	2015	State-of-the-art review on reproduction and pregnancy in rheumatic diseases.	Rheumatoid arthritis care during pregnancy must balance disease control and fetal safety.
Secher et al. [25]	2022	Found elevated risk of preeclampsia in rheumatoid arthritis, linked to disease activity and treatments.	Effective rheumatoid arthritis control may reduce preeclampsia risk.
Gupta et al. [26]	2010	Compared menstrual issues and pregnancy outcomes in rheumatoid arthritis and systemic lupus erythematosus.	Both diseases affect reproductive health; tailored care is crucial.
Peng et al. [27]	2025	Studied maternal and neonatal outcomes in autoimmune thyroid/primary Sjogren’s Syndrome pregnancies.	Rheumatoid arthritis and related autoimmune disorders increase pregnancy risks.
Barenbrug et al. [28]	2021	Examined tumor necrosis factor (TNF)-α inhibitor exposure during pregnancy in immune-mediated inflammatory diseases.	Anti-tumor necrosis factor drugs may be safe in pregnancy, with careful risk-benefit evaluation.

Here is a shorter version of the recommendations paragraph using clear medical terms: Women with RA should receive early preconception counseling, regular monitoring during pregnancy, and coordinated care from rheumatologists and obstetricians. Treatment should prioritize pregnancy-safe medications to maintain disease control while minimizing fetal risk. Patient education is essential to raise awareness of potential complications such as preterm birth and low birth weight. Postpartum care is equally important due to common disease flares, and guidance on medication safety during breastfeeding is recommended. Continued research is needed to improve pregnancy-specific RA management and outcomes.

## Conclusions

RA presents a complex and ongoing challenge to maternal and fetal health, especially for women with active disease or limited access to comprehensive care. The evidence indicates increased risks of complications such as preterm birth, low birth weight, and cesarean delivery among women with active RA. These risks are higher when the disease is not well controlled. Disease activity, especially during early pregnancy, emerges as a key predictor of adverse maternal and neonatal outcomes. Importantly, disease remission prior to and during pregnancy is strongly associated with better outcomes. Some women experience improvement during pregnancy, but symptoms often return after childbirth. While some RA treatments like methotrexate and certain JAK inhibitors raise concerns regarding teratogenicity, others appear safer when used appropriately under medical supervision. Furthermore, the safety and timing of immunosuppressive or biologic therapies during pregnancy remain critical concerns, requiring individualized care plans.
